# The Use of Larval Debridement Therapy and Negative-Pressure Wound Therapy for an Infected Wound After Thyroidectomy—A Case Report

**DOI:** 10.3390/jcm14165634

**Published:** 2025-08-09

**Authors:** Jolanta Dynarska, Edyta Zagrodnik, Agnieszka Kisielska, Anna Jurczak, Tomasz Machałowski, Sylwia Wieder-Huszla

**Affiliations:** 1 Medical Center Jolanta Dynarska, 70-953 Szczecin, Poland; rejestracja@dynarska-centrum.pl (J.D.); szpital@onkologia.szczecin.pl (A.K.); 2 Clinical Department of Anesthesiology and Intensive Care for Adults and Children, Pomeranian Medical University in Szczecin, 71-252 Szczecin, Poland; 3 Department of Specialized Nursing, Pomeranian Medical University in Szczecin, 71-210 Szczecin, Poland; anna.jurczak@pum.edu.pl (A.J.); sylwia.wieder.huszla@pum.edu.pl (S.W.-H.); 4 Department of Perinatology, Obstetrics and Gynecology, Pomeranian Medical University in Szczecin, 71-252 Szczecin, Poland

**Keywords:** larval debridement therapy, fistula, healing, wound, necrotic tissue, wound therapy

## Abstract

**Background:** Larval debridement therapy is used to cleanse necrotic tissue wounds and/or decontaminate wounds that are not amenable to standard therapies. **Methods:** A patient was diagnosed with septic shock and multiple organ failure caused by Streptococcus pyogenes after thyroidectomy (the patient had experienced contact with a child with scarlet fever six days before admission to the hospital). As a result of systemic infection, numerous necrotic skin lesions appeared, which involved the surgical site, chest and scalp. A tracheocutaneous fistula was confirmed. Due to the ineffectiveness of typical therapy and the patient’s severe clinical condition, she qualified for unconventional therapy (larval debridement therapy). **Results:** Larval wound debridement therapy and negative-pressure wound therapy were used for the tracheocutaneous fistula; this is the first case of this alternative therapy being described in the English-language literature. In this case, based on an analysis of the health benefits for the patient and the uncertain prognosis, larval therapy was used for a postoperative wound after strumectomy with the presence of a tracheocutaneous fistula, and negative-pressure wound therapy ultimately led to complete wound healing. **Conclusions:** Sepsis caused by Streptococcus pyogenes can be fulminant and often leads to complications or death, especially if it develops in the perioperative period. Larval therapy can be effectively used in cases of fistulas, such as tracheocutaneous fistulas, to prepare the wound bed for the next stage of healing using negative-pressure therapy, which ultimately leads to complete wound healing.

## 1. Introduction

For many years, larval debridement therapy (LDT) has been a well-established method of wound care in outpatient and inpatient settings, both in Poland and worldwide [1]. Its origins date back to the 1930s, although it has gained renewed/wider interest since 2010, when the larva of the green blowfly *(Lucilia sericata)* was described as an excellent microsurgeon, demonstrating the ability to carefully and selectively cleanse necrotic tissue wounds, eliminate wound infections and keep healthy tissue intact [2]. In Poland, LDT is used to cleanse necrotic tissue wounds and/or decontaminate wounds that are not amenable to standard therapies. The therapy is carried out using two basic maggot dressing designs: confinement dressings, which confine the larvae to the wound bed and allow them complete access to the wound; and containment dressings, where the maggots are contained in a net bag (a so-called BioBag), which facilitates their use but does not allow full access to the wound. The contraindications are listed in Table 1 [1,3].

According to the literature, the most common obstacles to the use of LDT are problems with the availability of larval therapy in public healthcare facilities, hospital protocols, psychological barriers and the negative attitudes of medical staff towards LDT [4]. The undoubted benefits of the therapy include a shorter wound healing time compared to other treatment methods and greater cost-effectiveness compared to hydrogel dressings, which translates into economic benefits. Such therapy is also a good option when there is no response to conventional treatment [5]. During the process of wound cleaning, the larvae secrete enzymes that destroy pathogenic microorganisms, and the movement of maggots promotes granulation, speeding up the healing process [6].

NPWT is a method that promotes wound healing; it involves the use of controlled negative pressure, which accelerates tissue regeneration, reduces the risk of infection and supports the process of angiogenesis. This method has been continuously improved and is gaining widespread use in surgery, chronic wound management and the treatment of complex wounds, with promising final results [7]. It is used in the treatment of chronic, surgical and traumatic wounds, as well as in skin and organ fistulas. Negative pressure generated by a specialized device that removes excess exudate reduces the number of bacteria in the wound and stimulates granulation [8]. Indications for and contraindications against NPWT are shown in Table 2.

Studies available in the literature indicate the effectiveness of this method in difficult, atypical cases involving the head and neck area and the thorax. The team of K. Ściskała described cases of patients in whom the use of NPWT allowed them to reduce effusion and accelerate the healing process [9].

## 2. Case Presentation

Patient X.Y. was 45 years old and hospitalized at the Department of Intensive Care Medicine at the University Hospital, where she was transferred from the Department of Plastic Surgery after the revision of the postoperative bed, following a previously performed thyroidectomy due to *Carcinoma papillare*, which was confirmed by histopathological examination. On the second postoperative day, the dynamic deterioration of the patient’s general condition was observed, with tachycardia, hypotension and desaturation despite passive oxygen therapy, as well as clinical features of shock with respiratory and circulatory failure, renal failure and biochemically increasing inflammatory parameters.

Due to cyanosis in the face, neck and chest areas, urgent computed tomography angiography (CTA) of the chest was performed with a pulmonary embolism protocol. It excluded pulmonary embolism but showed bilateral pleural effusion, suspected pneumonia and mediastinitis. In the operating theater, a sudden cardiac arrest occurred. Resuscitative measures were undertaken in accordance with the current algorithm, and hemodynamic heart function was restored. After the procedure, the patient did not awaken and was transferred to the intensive care unit (ICU). On admission, she was in a very serious condition, experiencing septic shock with multiple-organ failure. Intensive treatment and the monitoring typical of ICU conditions were implemented. The result of a microbiological test confirmed a positive wound culture (*Streptococcus pyogenes*).

The patient’s family history was clarified and completed; it was established that the patient had had contact with a child suffering from scarlet fever six days before admission to the hospital. During her stay in the ward, papular-type lesions with erythema and exfoliation of the epidermis in the course of streptococcal toxemia were observed on the skin, as well as necrotic lesions with epidermolysis and blisters locally on the chest. Skin and mucous membranes were typical of scarlet fever.

**Figure 1:**
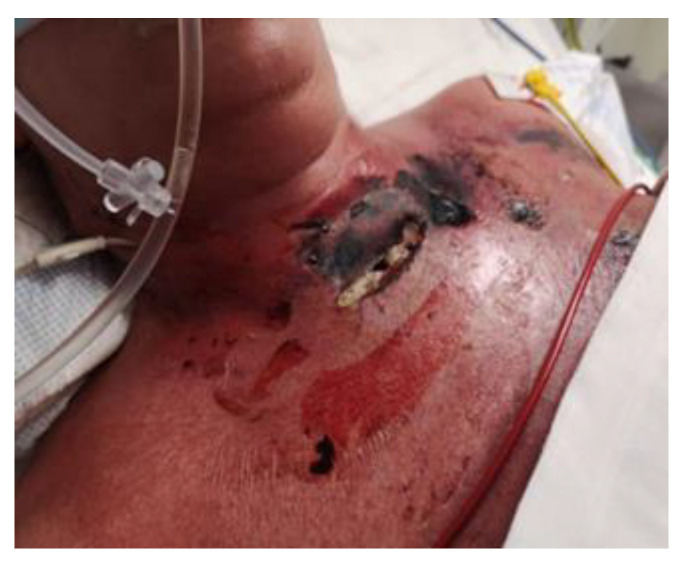
Postoperative wound with foci of necrosis and tracheocutaneous fistula.

As a result of systemic infection, numerous necrotic skin lesions appeared, involving the surgical site, chest and scalp. At the bottom of the wound, to the left of the trachea, a hole with leakage of mucous content was visible. A tracheocutaneous fistula was suspected and confirmed in an imaging study (Figure 1). The patient was assessed by a thoracic surgeon, surgeon and laryngologist. In the microbiological examination of the respiratory tract washings, *Pseudomonas aureginosa* was isolated. Table 3 is provided to facilitate an understanding of the course of the disease and the measures taken. It also includes the diagnosed infections and prescribed antibiotics at each stage of therapy. The main research question was whether it is possible to close a tracheocutaneous fistula when conventional treatment options have been exhausted.

**Figure 2:**
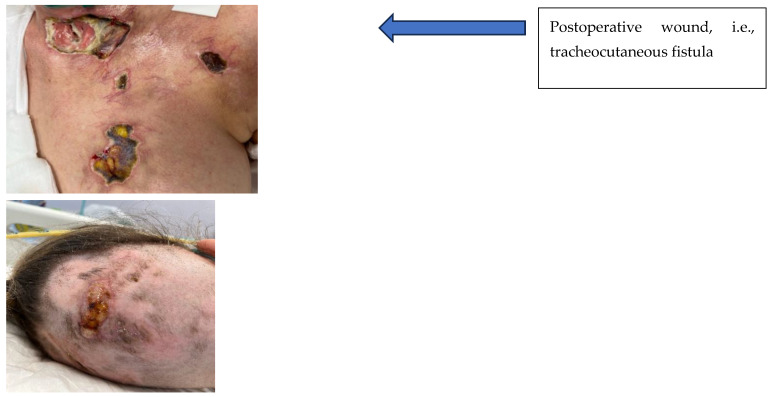
Chest ulcers and wound after thyroidectomy; a stage II pressure ulcer of the occiput―the first visit.

Due to the thinned, bacterially infected and necrotic skin of the neck, with a risk of anterior neck wall prolapse, the patient was assessed by a highly specialized wound care and dressing specialist, who confirmed the presence of wounds with intense local inflammation, fibrosis, necrosis and a visible biofilm firmly embedded in the tissue. The wounds in the chest area on the left side measured 4 × 5 cm and 2 × 1 cm. On the surface of the epigastrium in the area after the drain, a visible scab and inflammation were found. In the postoperative wound after strumectomy, a fistula with a diameter of 1.5 cm was observed, with the anterior wall of the trachea exposed. A large area of necrosis was present, with visible fibrin. There was an odor of medium intensity and abundant greenish brown exudate. A European Pressure Ulcer Advisory Panel/National Pressure Injury Advisory Panel (EPUAP/NPIAP) stage II decubitus ulcer was present on the occiput (Figure 2). The patient was in a moderate-to-severe general condition and was conscious and breathing independently (efficient self-breathing), periodically with oxygen supply. After physical and subjective examination, a treatment plan was established, and the patient’s consent to the proposed treatment was obtained. She was qualified for unconventional therapy (larval debridement therapy) after the prior preparation of the skin with the recommended dressings and preparations (Granudacyn liquid and gel antiseptics, Iruxol Mono, Granuflex). Initial wound preparation—i.e., wound necrectomy—was performed. The patient was fully aware of the seriousness of her clinical condition. Thanks to the painkillers that she was taking, she did not experience any pain. The patient, her husband and her family were kept informed and fully accepted the proposed treatment. She did not hesitate to decide on an alternative treatment due to the seriousness of her clinical condition and the risk of permanent damage to her health. All medical procedures were approved in writing by the informed patient.

The first step was to carry out the following:

- Reintubation of the patient to protect the respiratory tract from the entry of larvae defensins and to enable the suctioning of secretions (a protective measure in the event of the unintentional entry of secretions during LDT). An endotracheal tube with a tightly filled balloon located below the fistula formed a barrier between the respiratory system and the atmospheric air and the wound environment. The pressure in the ventilation tube balloon was measured regularly to avoid excessive increases, which would be dangerous for tracheal function and could cause necrosis. During treatment, the patient was reintubated and under analgesic sedation. She did not experience any discomfort from the treatment.
- The use of a BioBag in the area of the tracheocutaneous fistula (in larval therapy, this prevents the migration of larvae into the respiratory system).
- The implementation of NPWT after LDT. Since the bottom of the fistula was partially missing and leakage was possible, three-quarters of the fistula surface was lined with a silicone dressing with a large layer of solid silicone and small holes in the mesh structure. The surface of the bottom itself was covered from the outside with a small layer of a thin hydrocolloid dressing, which was glued to the silicone mesh to form a bottom in the wound bed. In addition, the wound was filled to its depth with a sterile polyurethane foam, and an external dressing was placed in the last layer.
- Next, treatment with targeted dressings was applied.

The following areas of attention were established:

- The trachea is a fairly rigid structure that is poorly supplied with blood, so there was a risk that it would become narrower after healing.
- In cases of infection, it is not possible to close the fistula (when the trachea is separated into two parts after purification via LDT)—consultation with an ENT oncologist ruled out the possibility of implanting a part of the trachea.
- If defensins flowed into the bronchi and lungs during LDT, the patient would require intensive monitoring.
- It is necessary to ensure the tightness of the NPWT when creating an artificial bottom.
- Wound treatment was considered according to the TIMERS strategy: tissue debridement (T), infection (I), moisture (M), epidermization stimulation (E), regeneration (R) and social and patient-related factors (S).

The patient and the staff caring for her were educated on wound healing issues. It was recommended to use nutritional products that would immunostimulate wound healing (Immuven two times a day), as well as to control the levels of inflammatory parameters and determine the level of vitamin D. Granudacyn liquid and gel antiseptics were applied, Iruxol Mono ointment was prescribed for wounds with a bottom and necrotic tissue, a Granuflex dressing was applied and the postoperative wound area was recommended to be moistened with physiological saline.

**Figure 3:**
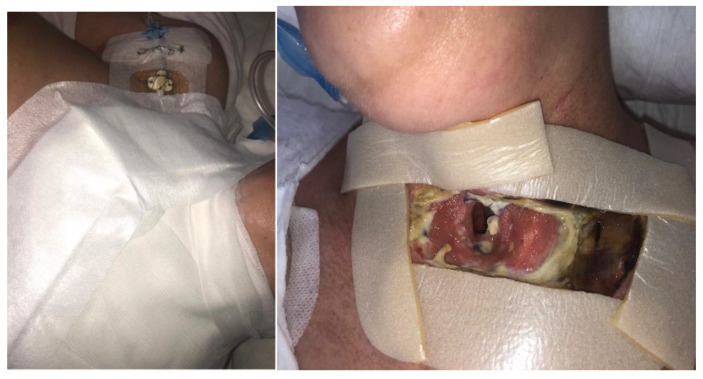
The wound prepared for larval dressing―the second visit (pre-LDT day).

At the second visit, the physical examination revealed persistent wounds on the chest―two wounds in the armpit and left breast in the debridement phase, with considerable local inflammation, necrosis and fibrin, and with a biofilm on the tissue surface. A wound was present in the postoperative area after thyroidectomy with a tracheocutaneous fistula, with sutures loosely placed in the area of the fistula opening after surgical debridement. A visible local wound infection was present with abundant exudate. The fistula opening was approximately 1.5 cm in diameter, with a visible endotracheal tube and the anterior wall of the trachea. There were numerous areas of necrotic tissue and fibrin, with a biofilm embedded in the tissue. After the lavaseptic treatment of the wounds with 0.9% NaCl, strips of Granuflex hydrocolloid dressing were applied, the wound edges were protected with zinc paste and a BioBag with larvae was applied (200 pieces from the Biolab culture). The larval dressing was secured with non-woven gauze pads and gauze strips. The wounds of the left thoracic region were prepared according to the TIMERS strategy. A large amount of material from the ulcers was obtained. Lavaseptics (0.9% NaCl), antiseptics (Granudacyn liquid―compresses for 15 min), Granudacyn gel, Iruxol Mono and Granuflex were applied (Figure 3).

During LDT, the condition of the dressings was monitored. The degree of wound debridement was checked and effective wound cleansing was achieved after five days of larvae therapy. A physical examination revealed the cleaned wound had subsiding local inflammation and a small biofilm; under the edges of the skin, there were small areas with fibrin, which were to be removed in the next stage. The remaining wounds had a small amount of necrotic tissue and biofilm, and around the wounds a visible decrease in inflammation was noted.

**Figure 4:**
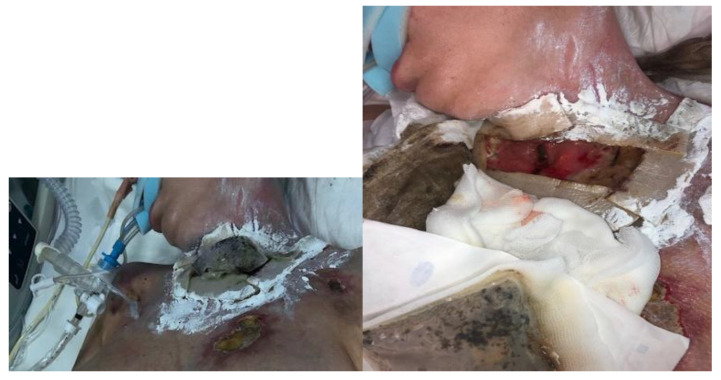

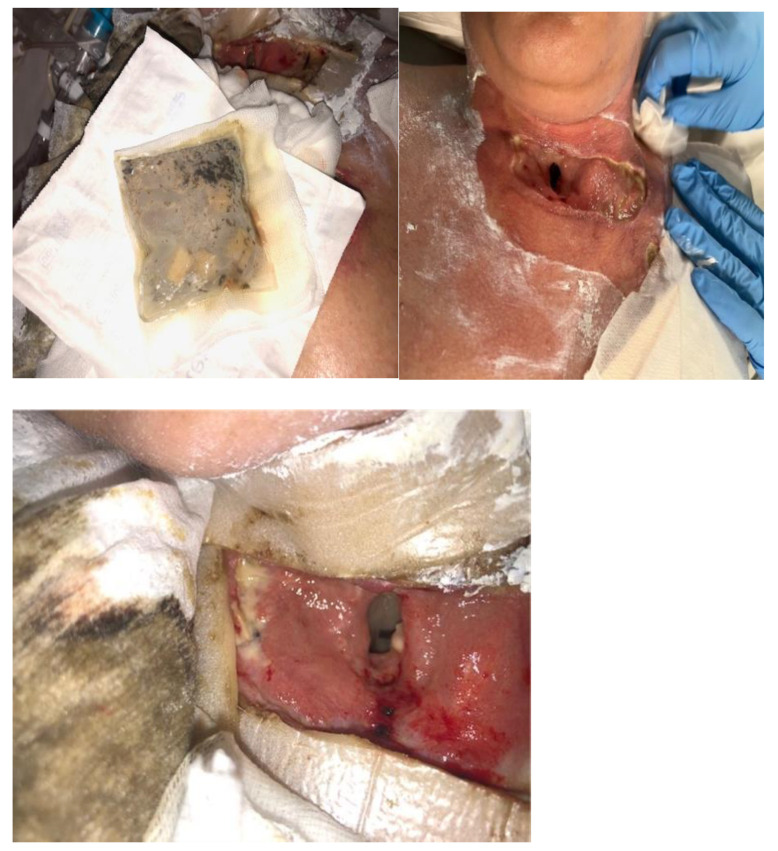
Removal of BioBag larval dressing and the degree of wound debridement―the third visit (post-LDT day).

**Figure 5:**
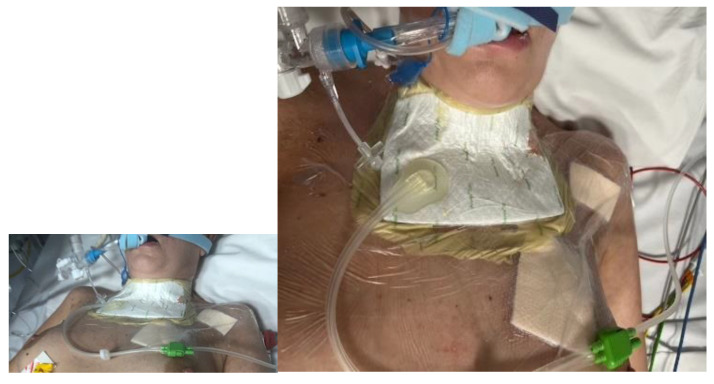
NPWT/Avance Solo.

**Figure 6:**
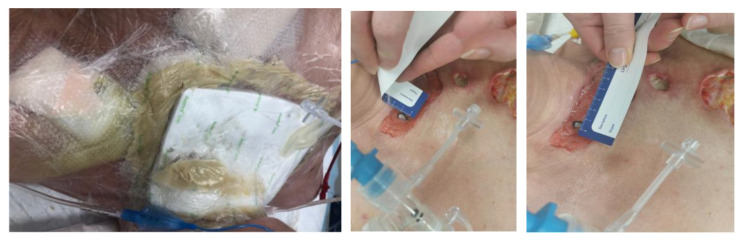

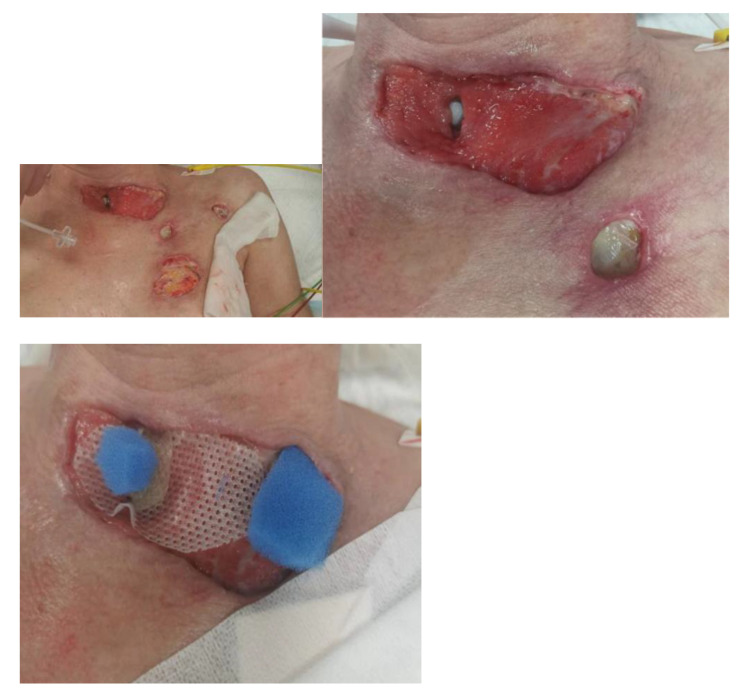
NPWT/Avance Solo (start of NPWT).

At the third visit (after 3 days), the BioBag with maggots was removed from the fistula (Figure 4). The wounds were prepared according to the TIMERS strategy under local anesthesia, and a small amount of material from the ulcers was obtained. Lavaseptics (0.9% NaCl), antiseptics (Granudacyn liquid―compresses for 15 min), Microdacyn gel, Aquacel Ag+ Extra dressings and Aquacel Foam were applied. Mepitel and Suprasorb H, of a similar size to the fistula, were applied to the area around the postoperative wound in the wound bottom. A sponge and the Avance Solo NPWT with a 15 × 20 cm dressing were placed in the area above the fistula. The system and dressings were applied for 7 days. A seal-tight dressing―stoma paste—was used for sealing (Figure 5). The patient was without fever, with normalized inflammatory parameters. During subsequent visits, the tracheocutaneous fistula decreased in diameter to approximately 0.5 cm × 1 cm (Figure 6).

**Figure 7:**
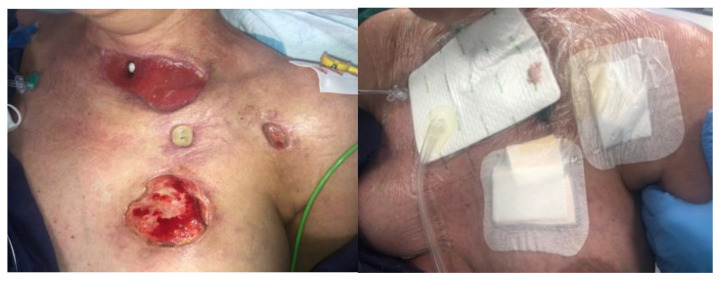

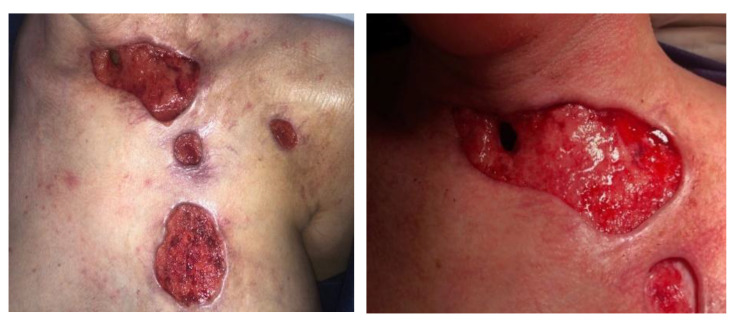
Completion of NPWT, improvement in ulcer condition—further treatment process continued by the ward staff.

During subsequent visits, the wound treatment plan was continued, achieving improvements in the condition of the ulcers, visible granulation tissue in the postoperative wound bed and the further gradual subsidence of inflammation. After four weeks of treatment, a physical examination revealed the presence of wounds with slight local inflammation, with visible granulation tissue and the presence of a biofilm (Figure 7).

**Figure 8:**
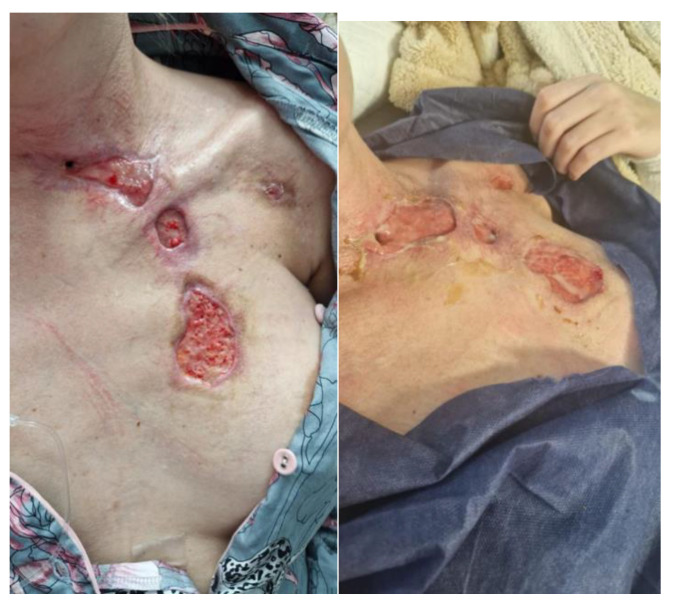
Continuation of treatment―the visible site of the fistula.

**Figure 9:**
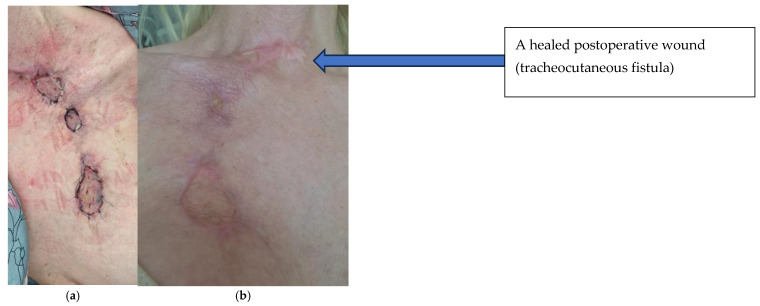
Image of the wound after four (**a**) and six (**b**) months—fistula wound healed spontaneously; other wounds closed via skin grafting (final follow-up).

After three months of intensive interdisciplinary treatment, the patient was transferred to the Department of Plastic Surgery for further treatment―the autotransplantation of skin (Figure 8). The fistula wound healed spontaneously, and other wounds were closed via skin grafting. The healing process and the appearance of the wound after 4 and 6 months are shown in Figure 9. In terms of long-term treatment outcomes, the patient has reported no respiratory complaints. The trachea is free of stenosis and has healed properly. The patient is in good condition, is fully independent and has no complaints. The patient was examined twice, after 12 and 18 months. The sites of fistula closure were practically invisible. There is no probability of symptom recurrence.

## 3. Discussion

Larval therapy is one of the most effective selective methods for wound cleansing and the reduction of bacterial loads and biofilms, which seem to be additional advantages of biosurgery in the era of increasing antibiotic resistance among microorganisms worldwide [10]. The debridement of wounds from necrotic tissue via this method occurs in a shorter period of time compared to the use of specialized dressings; it accelerates the process of tissue granulation, facilitating healing, and reduces the need for the systemic use of antibiotics in wound treatment [11]. Since the approval of larval therapy for wound treatment by the US Food and Drug Administration (FDA) in 2004, specialists have been using this biosurgery technique as an effective and economical solution for a wide range of wound etiologies, including diabetic foot disease, posttraumatic infections and necrotizing fasciitis, reporting beneficial treatment effects [12]. Guided by wound bed preparation (WBP) as one of the first elements of the TIMERS strategy, which is key in wound treatment, wound care specialists choose the most suitable method for the patient, seeking to eliminate necrotic tissue and stimulate the wound bed to initiate healing processes [13].

Contraindications to the use of larval therapy include the presence of wounds penetrating into body cavities, which is due to the technical inability to flush out the larvae after the therapy is completed; the risk of contact between sensitive tissues in body cavities and the irritating substances secreted by the maggots; and the inability to protect them [1]. The literature also lists wounds located in the area of the respiratory tract as contraindications [5]. In the discussed case, due to the patient’s condition, the exhaustion of wound treatment options using conventional methods, prolonged hospitalization and the uncertain prognosis, a therapeutic decision was made to use LDT to treat the postoperative wound after strumectomy with a tracheocutaneous fistula. A containment dressing with 200 larvae—the so-called BioBag—was used to cleanse the necrotic tissue after protecting the skin around the wound with zinc paste. The correct application of a maggot containment dressing and the lack of excessive pressure with secondary dressings protect against the displacement or rupture of the larvae sachet and facilitate the removal of the larval dressing at the end of therapy [1]. After being informed about the possible complications of LDT, the patient gave informed written consent to both the use of larval therapy and reintubation before starting the wound treatment. Throughout the application of the larvae, the wound remained under constant monitoring and observation with regard to the extent of debridement and dressing changes. It is considered that larvae in containment dressings feed less effectively than in confinement dressings. Due to the choice of form and the daily control of the wounds, the size of the maggots and the degree of wound cleansing, the application of the larval dressing was extended to five days [14]. Biosurgery with *Lucilla Sericata* larvae effectively cleaned the bottom of the wound bed and allowed treatment to continue with NPWT. This method proved effective in controlling exudate and promoting wound closure. However, the need for detailed monitoring of the patient was emphasized to avoid complications related to the possibility of air leakage.

There are no similar case reports of LDT/NPWT use in fistulas, especially respiratory tract fistulas. This is a pioneering solution, and represents the first case report in the English-language literature. Two case reports of LDT/NPWT use on a hand and wrist wound can be found in the literature, but they did not use a special BioBag to prevent larvae from escaping from the bag [15]. This highlights the innovativeness of our solution, especially given the proximity of the bronchial tree. A meta-analysis from 2024 [16] indicated that LDT is more effective in accelerating wound healing compared to NPWT. LDT appears to be the preferred method for the treatment of chronic ulcers in older patients, emphasizing the importance of efficient healing in improving patients’ quality of life [16]. The above articles highlight the innovativeness, relevance and clinical value of our treatment.

NPWT is an effective method of wound management in various difficult cases, including cutaneous and organ fistulas [8,17,18]. It can significantly shorten the hospitalization period, reduce the risk of infection and improve treatment outcomes. Although the use of NPWT in tracheocutaneous fistulas has not yet been widely described in the literature, the end result suggests its potential effectiveness.

## 4. Conclusions

Sepsis caused by Streptococcus pyogenes can be fulminant and often leads to complications and death, especially if it develops in the perioperative period. Larval therapy can be effectively used in cases of fistulas, such as tracheocutaneous fistulas, to prepare the wound bed for the next stage of healing using negative-pressure therapy (which was used for the first time in history in this clinical case). This ultimately led to complete wound healing. This method may be recognized in the future as the gold-standard treatment, but it requires further thorough research.

## Figures and Tables

**Table 1:** Contraindications to the use of larval therapy [1,3].

Contraindications to LDT
Lack of knowledge of the etiology of the wound
Necrosis and dry, scab-covered wounds
Wounds penetrating into body cavities, very deep wounds that may potentially communicate with body cavities
Severe coagulation disorders, active bleeding from the wound, wounds in the immediate vicinity of large vessels
Anemia requiring transfusion of blood products
Ischemic wounds without revascularization treatment
Presence of a tumor at the bottom or edges of the wound
Fever, infection spreading beyond the wound bed, systemic infection
Lack of patient acceptance of LDT as a treatment method, lack of patient consent for LDT, lack of cooperation from the patient
Allergic reactions to insect chitin, eggs, soy, or fly larvae
Pregnancy

**Table 2:** Indications for and contraindications against the use of NPWT [7,8].

Indications	Contraindications
Chronic wounds (bedsores, shin ulcers)	Dry and hard necrosis
Postoperative and posttraumatic wounds	Uncontrolled systemic infections
Infected and heavily exuding wounds	Fistulas leading to the cavernous organs
Skin and organ fistulas	Presence of tumor tissue in the wound
Diabetic foot disease	Uncontrolled bleeding

**Table 3:** Chronological clinical course of treatment.

Date	Medical Incident
5 January 2023	Operated on a scheduled basis—total strumectomy due to ca. papillare.
6–7 January 2023	Dynamic deterioration in general condition, hypotension, tachycardia and desaturation, requiring passive oxygen therapy. Dynamic septic shock after prior revision of the postoperative wound, treatment in the intensive care unit, broad-spectrum empirical antibiotic therapy: meronem + vancomycin + clindamycin + metronidazole.
9 January 2023	Right pleural drainage—approximately 400 mL of dark brown fluid was evacuated. Microbiological result: positive culture from the wound—*S. pyogenes*). Antibiotic therapy was modified (vancomycin and metronidazole were discontinued; ampicillin was added).
11 January 2023	The skin was entirely covered with papular eruptions, with erythema and exfoliation of the epidermis in the course of streptococcal toxemia, and with localized necrotic lesions with epidermolysis and blisters on the chest and neck.
16–18 January 2023	Candida albicans was identified in bronchial washings, and fluconazole and clotrimazole were administered topically. Thoracentesis was performed, evacuating 800 mL of fluid.
2–4 February 2023	A tracheal fistula was suspected, confirmed using a neck CT scan. In microbiology, Klebsiella pneumoniae sp. was identified on a vascular catheter and punctures were listed. Tazocin was administered. Swab from the wound: Pseudomonas aeruginosa MDR. Avibactam administered intravenously + colistin in nebulization.
11 February 2023	Consulted with a specialist in dressings and wound treatment. The patient was qualified for larval therapy on February 15, after prior preparation of the skin with the recommended dressings and preparations (Granudacyn liquid and gel antiseptics, Iruxol Mono, Granuflex).
15 February 2023	The patient was intubated. Preliminary treatment was performed: wound necrectomy and a dressing with 200 larvae was applied.
18 February 2023	Larval therapy was completed and NPWT was applied (−150 mmHg).
8 March 2023	NPWT therapy was completed, mechanical and chemical cleansing was applied to the wound, and dressings containing silver ions were applied. The patient was extubated and left on spontaneous breathing with slight oxygen supplementation.
7 April 2023	Planned skin autotransplantation procedure for granulation of wounds, leaving a free field above the tracheal fistula. Local wounds after strumectomy and necrectomy with secondary skin autotransplantation secured with an occlusive dressing.
July 2023/October 2023	No complications. Planned skin autotransplantation procedure for granulation of wounds, leaving a free field above the tracheal fistula. Local wounds after strumectomy and necrectomy with secondary skin autotransplantation secured with an occlusive dressing.

## Data Availability

Data are available on request from the first author.

## Abbreviations

The following abbreviations are used in this manuscript:

LDT	Larval debridement therapy
NPWT	Negative-pressure wound therapy
CTA	Computed tomography angiography
ICU	Intensive care unit
FDA	Food and Drug Administration
WBP	Wound bed preparation
